# London Journals—Creosote in Tooth Ache

**Published:** 1838

**Authors:** 


### Art. VII.—London Journals.

The Lancet, to the end of April is lying before us. A considerable part of each number is taken up with Lectures of the theory and practice of Medicine by Dr. Marshall Hall; and on Materia Medica and Therapeutics by Dr. Seigmond. We are obliged to say, that in looking through the numbers, we do not find much of novelty, either in theory or practice; but a great deal of controversy on the subject of Lectures, Hospitals, Medical police and Animal Magnetism—the last of which seems likely to become a topic of general interest and elaborate inquiry in London. It is worthy of remark, that this has not hitherto been the case in that city.

The Medical Gazette for April, presents us with Lectures by Dr. Southwood Smith, on Forensic Medicine; Dr. C. J. B. Williams, on Diseases of the Chest; and Dr. H. Clutterbrak on Blood-letting. The amount of strictly scientific and practical matter in the *Gazette*, is on the whole greater than in the *Lancet*. Among the papers is one by Edwin Saunders Esq., Surgeon on the "Employment of Creosote as an Odontalgic remedy," from which we shall quote a few passages.

"It is well known that the human teeth are exceedingly liable to fall into a morbid condition, in which a disorganization and decomposition of their structure take place, and which is familiarly recognized under the terms caries or gangrene. The origin, exciting causes, and precise nature of this disease, are still involved in much obscurity, and will probably long continue, as at present, to divide the opinions of physiologists. It is much less prevalent in the ruder and more uncivilized states of society, and almost unknown amongst the lower animals. It would seem, therefore, to be in some degree connected with a refined and artificial mode of existence, frequent indisposition, the free use of various drugs, and infantile diseases, which, occurring during the formation of the teeth, render their structure imperfect, and less capable of resisting morbid influences in after life. But whatever may be the causes of this very general affection, and probably if they could be clearly ascertained and defined, they would be found altogether beyond control, the effects are uniformly and unequivocally injurious.—Commencing by a small dark spot immediately under the enamel, which soon breaks down, from losing the support of the subjacent bone, portion after portion of the osseous structure of the tooth is destroyed, until the central cavity and its contained vessels become exposed. In this state the contact of atmospheric air, of the tongue, or the slightest pressure of a small particle of food on the delicate and extremely vascular structure thus suddenly denuded, is sufficient to excite in it a high degree of inflammation. From the circumstances of the case, however, being included within a circumscribed cavity whose hard unyielding walls admit of no swelling or expansion of the highly organized membrane it contains, an intensely congested state of the vessels and nerves takes place, and produces that hell o' a' diseases, as Burns has emphatically styled it, the toothache. In these cases, antiphlogistic treatment is out of the question, from the difficulty of its application; and even in the few cases in which it is practicable, as I have more than once experienced, fails to afford the slightest relief. The only treatment of which such cases appear susceptible, and to which, if judiciously applied, the symptoms almost invariably yield, frequently in a very short space of time, is the reiterated application of some highly stimulating or narcotic drug. Amongst the most accessible and valuable preparations of this kind is the camphorated spirit; it is generally far more efficacious than the tinctura opii, lunar caustic, and many other of the popular remedies usually amployed in such cases. The mineral acids, which sometimes afford a temporary relief, are highly objectionable, on account of their action upon the surrounding bone, which speedily destroys the tooth, re-exposes the membrane, and gives rise to a fresh, and probably still more severe attack of the pain. It is in these cases, however, that the creosote becomes valuable, and probably has a good claim to be considered a specific. In few cases of pure toothache, arising from exposure of the internal membrane, not complicated with inflammation of the periosteum, will it fail, after two or three applications, to afford relief. To obtain its best effects, however, some care is required in its application. Fremanger, in the Bulletin Général Thérapeutique, has laid much stress upon the importance of previously wiping out the cavity with a small piece of lint introduced upon the point of a probe: and whenever this is practicable, it is undoubtedly a desirable preliminary, because the mucus of the mouth, which is always abundant where the masticatory function is suspended, will prevent the application from coming immediately into contact with the diseased structure. In a vast number of cases, however, the patient will be utterly unable to endure the agony which this preparatory process inflicts, even when performed in the gentlest manner. A more important hint, but which is too seldom regarded, is to employ the cotton or lint which serves as the vehicle for its introduction, in as small a quantity as possible. The more common practice is to introduce a piece of lint or wool of sufficient size to fill the cavity, which requires so much pressure for its insertion as to cause it to part with a considerable portion of the drug, and thus to prevent it reaching the seat of the disease. If relief be not obtained at once, fresh applications may be made every ten or fifteen minutes, until some effect appears to be produced. Care should also be taken that the wool or lint be not too fully charged with the creosote, considerable pain and inflammation being frequently the result of the effusion of a small quantity on the gums and lips. Mr. Cormack observes, "I once saw a pretty extensive sore, resembling a burn in appearance, caused by the overflow of saliva, which ensued upon the application of creosote to a carious tooth." With the utmost care, however, it is scarcely possible to prevent a small portion escaping into the mouth, more especially if the tooth be situated in the upper row; but this, if mixed with the saliva, which generally flows in great abundance on its application, will occasion but little inconvenience. If the diseased tooth be situated in the lower row, the effect will generally be obtained in a much shorter time than in the upper, because in the former case the creosote will tend towards the seat of disease by its own gravity, while in the latter, unless the patient be placed in a recumbent posture, that force will exert a contrary influence. It is an useful hint to make the patient recline with the head low, and on that side in which the affected tooth exists; as, for instance, if the application be made to one of the molar teeth, on the right side of the upper row, direct the patient to lie on the right side for a short time; the creosote will thus be likely to reach the affected spot, and the risk of any portion of it passing into the throat and stomach will thus be obviated. It would be scarcely necessary to observe, were it not that the notion has been entertained by some of its more zealous advocates, that creosote can exert no preservative effect upon the tooth itself. It simply alleviates the symptoms, without in any degree arresting the progress of the caries, for which different treatment must be sought. The opinion, however, which has been held by others, that it acceleretes the decomposition of the bony structure of the tooth, seems to be equally unfounded, since neither ankaline nor acid qualities are developed on the application of the most delicate tests.

